# Development of the Disturbed Eating Characteristics Scales (DECS)

**DOI:** 10.1186/2050-2974-2-S1-P11

**Published:** 2014-11-24

**Authors:** Kim Woodward, Doris McIlwain, Alan Taylor

**Affiliations:** 1 Macquarie University, Sydney, Australia

## 

Why is another measure of disturbed eating necessary? While there is a growing literature identifying links between certain emotion regulation styles and eating disturbances, much contention and contradictory evidence exists regarding the distinction between different specific eating disorder (ED) diagnoses in such relationships. This is possibly due to the common and overlapping features of different ED diagnoses and rather what is needed is exploration of the link between certain emotional styles and specific ED characteristics. However, in order to explore such relationships, a freely available self-report measure that reliably and validly identifies specific characteristics of eating disturbances is required. The current study aimed to develop such a measure. To this end an initial 66-item pool was devised and tested in Study 1 with a sample of 403 women. An exploratory factor analysis revealed a four-factor structure retaining 59 items. A confirmatory factory analysis (CFA) supported this factor structure and lead to the identification of four sub-scales 'Drive for thinness' (31-items), 'Binge' (10-items), 'Purge' (9-itmes), and 'Extreme-restraint' (9-items). These sub-scales were found to have high internal consistency and good reliability and validity. In Study 2 this factor structure was again found in a CFA with a sample of 749 women.

